# A novel tool for predicting the survival of endoprosthesis used for reconstruction of the knee following tumor resection: a retrospective cohort study

**DOI:** 10.1186/s12885-021-08710-x

**Published:** 2021-09-03

**Authors:** Cheng-gang Pang, Xiong-gang Yang, Yun-long Zhao, Yan-cheng Liu, Yong-cheng Hu

**Affiliations:** 1 Graduate School, Tianjin Medical University, Tianjin, 300070 China; 2 Department of Orthopedic Surgery, Huashan Hospital, Fudan University, No. 12 Wulumuqi Road, Shanghai, 200040 China; 3 Department of Bone Tumor of Tianjin Hospital, Tianjin, 300211 China

**Keywords:** Limb salvage, Prognostic factor, Tumor endoprostheses, Nomogram

## Abstract

**Background:**

Prosthesis-related complications, after knee reconstruction with endoprosthesis during operation for tumors around the knee, remain an unresolved problem which necessitate a revision or even an amputational surgery. The purpose of the current study was to identify significant risk factors associated with implant failure, and establish a novel model to predict survival of the prosthesis in patients operated with endoprostheses for tumor around knee.

**Methods:**

We retrospectively reviewed the clinical database of our institution for patients who underwent knee reconstruction due to tumors. A total of 203 patients were included, including 123 males (60.6%) and 80 (39.4%) females, ranging in age from 14 to 77 years (mean: 34.3 ± 17.3 years). The cohort was randomly divided into training (*n* = 156) and validation (*n* = 47) samples. Univariable COX analysis was used for initially identifying potential independent predictors of prosthesis survival with the training group (*p* < 0.150). Multivariate COX proportional hazard model was selected to identify final significant prognostic factors. Using these significant predictors, a graphic nomogram, and an online dynamic nomogram were generated for predicting the prosthetic survival. C-index and calibration curve were used for evaluate the discrimination ability and accuracy of the novel model, both in the training and validation groups.

**Results:**

The 1-, 5-, and 10-year prosthetic survival rates were 94.0, 90.8, and 83.0% in training sample, and 96.7, 85.8, and 76.9% in validation sample, respectively. Anatomic sites, length of resection and length of prosthetic stem were independently associated with the prosthetic failure according to multivariate COX regression model (p<0.05). Using these three significant predictors, a graphical nomogram and an online dynamic nomogram model were generated. The C-indexes in training and validation groups were 0.717 and 0.726 respectively, demonstrating favourable discrimination ability of the novel model. And the calibration curve at each time point showed favorable consistency between the predicted and actual survival rates in training and validation samples.

**Conclusions:**

The length of resection, anatomical location of tumor, and length of prosthetic stem were significantly associated with prosthetic survival in patients operated for tumor around knee. A user-friendly novel online model model, with favorable discrimination ability and accuracy, was generated to help surgeons predict the survival of the prosthesis.

**Supplementary Information:**

The online version contains supplementary material available at 10.1186/s12885-021-08710-x.

## Background

The distal femur and the proximal tibia are common sites for primary and metastatic bone tumor [1]. Amputation was the traditional surgical treatment for musculoskeletal sarcomas of the extremities, with the aim of saving the patients’ life. With the simultaneous improvement on the understanding of the biology and staging of tumors, reconstructive techniques, and effective adjuvant chemotherapy, however, limb salvage surgery has replaced the amputation as the predominated treatment choice for more than 90% of patients with a primary malignancy on the knee [2–4].

Because of the unique biological inherent properties of the tumor, the complex anatomic features of the knee and problems associated with extensor reconstruction, tumors arising around the knee pose a unique set of challenges in the management of soft tissues and the restoration of limb length and joint kinetics. The options for reconstruction after resection of a tumor around the knee joint include implantation of a prosthesis, osteoarticular allograft, allograft-prosthesis composite, recycled autologous bone graft, arthrodesis with intercalary bone grafting or conversion to a rotationplasty [5]. Among these methods, tumor prosthesis offers the advantages of convenience, early mobilization, and weight bearing capability, and it allows the early introduction of postoperative adjuvant therapy. Furthermore, there are no differences in survival rates between limb-salvage and ablative surgery [6–8].

Although advances in materials and implant design have occurred over the past three decades, complications and failures of these devices remain high compared to other arthroplasty procedures [9–11]. Prosthesis-related complications, including infection, aseptic loosening, dislocation, recurrence of the tumor, joint stiffness or contracture, instability of components, and implant mechanical weaknesses, remain an unresolved problem which necessitate a revision or even an amputational surgery [12–14]. High rate of prosthesis-related failure indicates that tumor endoprostheses need to be further studied, and improvements in surgical technique and component design are required. To guide the treatments selection and decision making, it may be crucial to clarify the prognostic factors that significantly predict the survival of prosthesis used for reconstructing the knee operated for tumors. Until now few studies have analyzed the prognostic effect of patients’ and operation-related characteristics.

The nomogram is a type of easy-to-use predicting model, which transforms traditional statistical predictive models into visualized probability estimates tailored to each patient. Using this model, one could be provided with continuous survival probabilities at several time points that predicted for an individual patient. What is more, the nomogram could be further transformed into a webpage based predicting widget, easily providing a predicted Kaplan-Meier survival curve, and survival rate at each time point.

Thus, the purpose of this study was (1) to evaluate the clinical and functional outcomes of patients who underwent a limb salvage surgery for tumors around the knee, (2) to identify prognostic factors significantly associated with implant failure, (3) and to develop a validated nomogram model as well as a webpage based predicting tool.

## Methods

This study was conducted according to the Transparent Reporting of a Multivariable Prediction Model for Individual Prognosis or Diagnosis (TRIPOD) statement [15], and reported according to the Strengthening the Reporting of Observational Studies in Epidemiology (STROBE) Statement (Available in Additional file 1).

### Patients inclusion

In accordance with the Declaration of Helsinki and upon the attained ethical approval from the hospital ethics committee, information of 297 consecutive patients with neoplastic disease around the knee who were treated with tumor resection and subsequent endoprosthetic reconstruction between April 1995 and August 2018 in two individual research centers were retrospectively reviewed for potential eligibility.

Patients would be included following the inclusion criteria: (1) age ≥ 14 years at the time of surgery; (2) bone tumors located around the knee; (3) limb-salvage or ablative surgery. The initial diagnosis was made based on pre-operative radiographs (X ray, magnetic resonance imaging [MRI] or computed tomography), and consequently confirmed by core needle biopsy or by open biopsy and pathological examination. Patients with reconstructed knee for medical conditions other than bone tumor, patients lost to follow-up, or patients with incomplete medical document were excluded.

### Surgical treatment

All patients indicative for limb-salvage surgery were evaluated systemically on the lesion size, preoperative planning of the level and optimal types of prostheses. The dimensions of the tumor and boundaries were measured by a combination of total leg-length radiographs and cross-sectional imaging, including MRI or 3D Multi-Slice Computerized Tomography (3D MSCT). With the completion of imaging materials and histology diagnosis, patients were categorized to a surgical stage according to Musculoskeletal Tumor Society (MSTS) classification [16] and received neoadjuvant chemotherapy on protocol when applicable. After resection of the tumor, reconstruction was performed using a cemented metal endoprosthesis (custom/modular). During the early time of the surgery, custom implants (fixed/hinged) were used in 88 patients. Modular implants featuring a rotating hinge mechanism were subsequently implanted in the remaining 63 patients. The surgical resection and reconstruction were performed by two senior professors, whose members of the team were orthopedic surgeons with extensive experience in the field the knee arthroplasty procedures, following the generally accepted oncologic principles of obtaining a wide surgical margin. Excision was performed through a medial or lateral parapatellar approach. Frozen section analysis of the intramedullary canal contents at the resection site was performed intraoperatively to ensure a negative marrow margin. An intraoperative radiograph was also performed to confirm that the fine line force of the knee was obtained for all the patients. We then performed a soft tissue reconstruction for each individual to achieve muscular coverage of the implants and to restore function of the knee.

Post-operative management mainly focused on prophylaxis of incision infection and application of antithrombotics and analgesics. Intravenous antibiotic prophylaxis was maintained until the wound drains were removed. Mobilization commenced on the second day postoperatively with the assistance of a walker with the purpose of preventing thrombogenesis or development of a infection in the urinary systems.

### Post-operative rehabilitation protocol

In order to obtain the maximum function of the knee postoperatively, detailed procedures were described as follows. Firstly, immediate weight bearing assisted with the hinged brace around the knee for all patients was permitted due to the stability of the cemented implants and the stability of reconstructed knee. A continuous passive motion (CPM) apparatus was used under professional rehabilitation therapist’s guidance and supervision on the first day post-operatively and continued until discharge. Extra attention was paid for patients with reconstructions of the extensor mechanism, whose motion was commenced from − 5° to 10° extension to 30° to 45° flexion and gradually increased to greater than 90° flexion before discharge. When rehabilitation was executed, patients were administrated appropriate anodyne, which allowed them an indolent restoration.

### Recorded variables

The patients were required to be interviewed every 3 weeks for the first 3 months after surgery, then on a quarterly basis for 3 years, semiannually for an additional 2 years, and then annually. The medical records, radiographs and pathologic records, operation details, information about adjuvant oncological therapies, and oncologic outcomes were reviewed. We recorded the following variables as the potential prognostic factors: age at operation, sex, body mass index (BMI), tumor types (divided into borderline tumor, malignant primary tumor, and metastatic bone tumor), operation related complications within 6 months, adjuvant chemotherapy/ radiotherapy, pathological fracture, primary/ revision reconstruction, anatomical location (proximal tibia or distal femur), site of knee (left or right), bleeding volume, operation time, bone transplantation, prosthesis type (custom or modular), prosthesis motion mode (rotational or fixed), length of prosthetic stem, diameter of prosthetic stem, material of prosthesis (titanium or vatallium), and length of resection.

The outcome variable was prosthesis survival, which was defined as the time interval between operation and prosthesis failure (any cause that required replacement of the components of the prosthesis).

#### Statistical analysis

The included patients were randomly divided into training sample and validation sample with a ratio of 7:3. The training sample was used for model establishing and internal validation, while the validation sample was used for external validation. The continuous variables, including the age, BMI, length of prosthetic stem, diameter of prosthetic stem, length of osteotomy, operation time, and bleeding volume, were transformed into dichotomous variable using the cutoff point detected with the method of running log rank test [17, 18]. The distribution of the patients according to each prognostic factor was described as mean ± SD or percentage. Kaplan-Meier survival curve was used to describe the overall patients’ survival and prosthetic survival, in training and validation samples.

Univariate analysis using COX proportional hazards model was performed to initially identify the potential significance of each prognostic variable on predicting the prosthetic survival. All of the variables demonstrated to be significantly or marginally significantly (*p* < 0.15) associated with prosthetic survival were included in the multivariable COX analysis. Kaplan-Meier survival curves were generated for all prognostic factors involved in multivariable analysis. Hazard ratio (HR) and confidence interval (95%CI) were selected as the effect size for both univariate and multivariate COX model. A forest plot was generated to display the result of multivariate analysis.

Basing on the significant variables screened by multivariate COX model, a novel nomogram was plotted, to graphically provide continuous survival rates at several time points and the median survival time of prosthesis. C-index and calibration curve were used to evaluate the discrimination ability and accuracy of the nomogram respectively. Finally, after fitting of a new COX model using the variables involved in the graphical nomogram, a webpage-based dynamic nomogram, which could provide predicted survival curve and survival rate at any time point as wanted, was established.

All analyses have been performed with program of R language (Foundation for Statistical Computing, Vienna, Austria). The univariable and multivariable COX models were fitted using the R package “survival”. The graphical nomogram and the online dynamic nomogram were generated with the packages “rms” and “DynNom”. Level of statistical significance was set at *P* < 0.05.

## Results

### Patients’ baseline characteristics

**Fig. 1:**
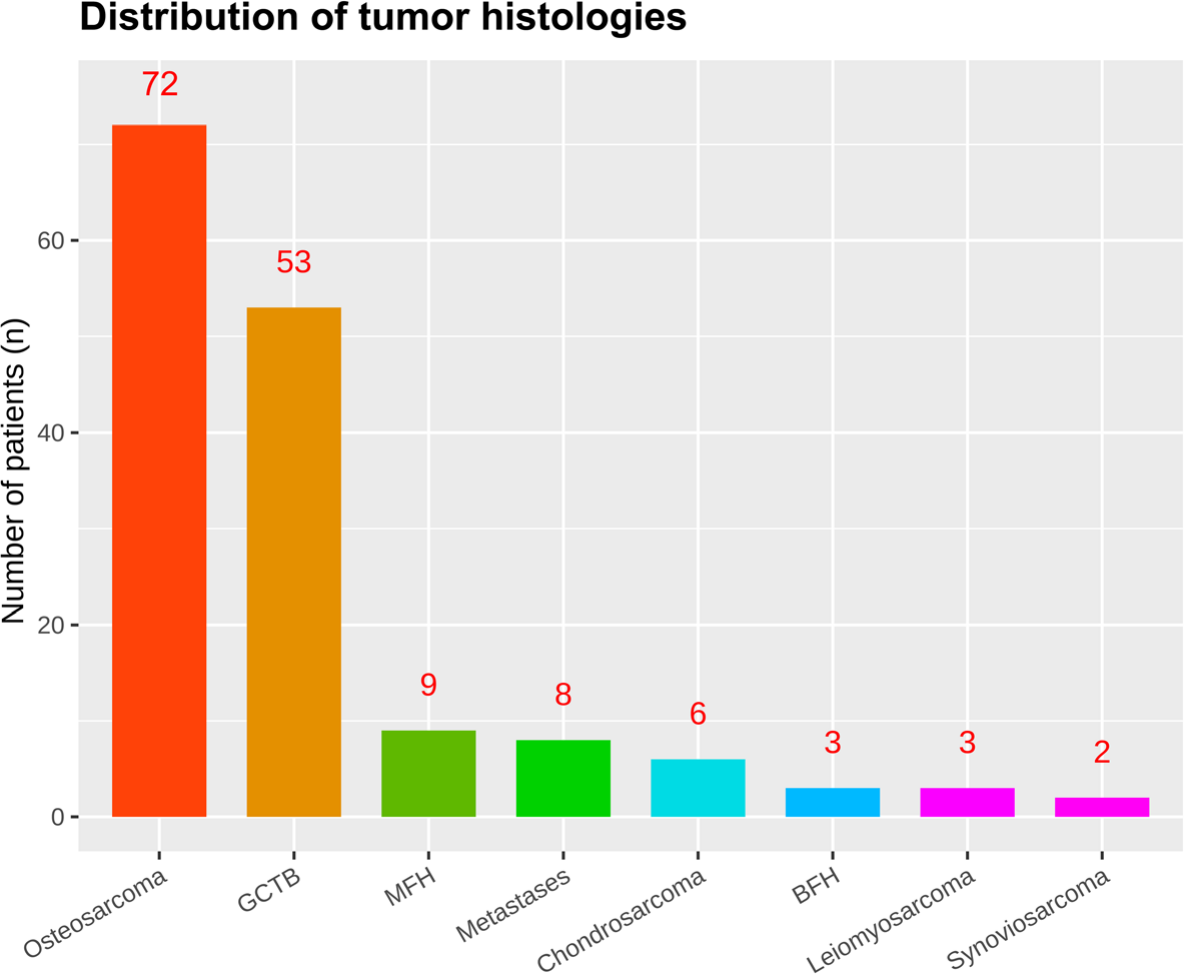
Distribution of the tumor histology. Abbreviations: GCTB, giant cell tumor of bone; MFH, malignant fibrous histiotoma; BFH, borderline fibrous histiotoma

A total of 203 patients (156 divided into the training sample and 47 divide into the validation sample) were eligible for final inclusion according to the inclusion and exclusion criteria. The baseline characteristics is shown in the Table 1. Among these patients, there were 99 males (63.46%) and 53 (36.54%) females, and 24 male (51.06%) and 23 female in the training and validation samples, respectively. The mean age was 35.31 ± 17.54 and 31.09 ± 16.17 years in the training and validation samples at the time of surgery. The distal femur (*n* = 99, 63.46%) was more common than proximal tibia (*n* = 57, 36.54%) concerning the involved anatomical location. The distribution of the tumor histology is presented in Fig. 1, showing that malignant primary tumor (osteosarcoma, 72; malignant fibrous histiotoma, 9; chondrosarcoma, 6; leiomyosarcoma, 3; synoviosarcoma, 2) was the predominated tumor type, followed by borderline tumor (giant cell tumor of bone, 53; fibrous histiotoma, 3) and malignant metastatic tumor.

**Table 1:** Summary of patients’ baseline characteristics

Variables	Training sample		Validation sample	
**Age**				
Average (n/mean ± SD)	156	35.3 ± 21.1	47	31.1 ± 16.2
< 58 years (n/%)	132	84.62	42	89.36
≥ 58 years (n/%)	24	15.38	5	10.64
**Sex** (n/%)				
Male	99	63.46	24	51.06
Female	57	36.54	23	48.94
**BMI**				
average (n/mean ± SD)	41	25.4 ± 3.9	11	27.8 ± 3.9
< 26.8 (n/%)	26	63.41	4	36.36
≥ 26.8 (n/%)	15	36.59	7	63.64
**Tumor types** (n/%)				
Borderline tumor	56	35.90	16	34.00
Malignant primary tumor	92	58.97	29	61.70
Malignant metastatic tumor	8	5.13	2	4.30
**Operation related complications** (n/%)				
No	130	83.33	40	85.11
Yes	26	16.67	7	14.89
**Adjuvant chemotherapy/ radiotherapy** (n/%)				
No	84	53.85	24	52.17
Yes	72	46.15	22	47.83
**Pathological fracture** (n/%)				
No	118	75.64	38	80.85
Yes	38	24.36	9	19.15
**Primary/ revision reconstruction** (n/%)				
Primary reconstruction	112	71.79	36	76.60
Revision reconstruction	44	28.21	11	23.40
**Anatomical location** (n/%)				
Distal femur	99	63.46	31	65.96
Proximal tibia	57	36.54	16	34.04
**Site of knee** (n/%)				
Left	85	54.49	25	53.19
Right	71	45.51	22	46.81
**Bleeding volume** (n/%)				
average (n/mean ± SD)	144	790.4 ± 602.6	34	597.7 ± 389.1
< 1000 ml	97	67.36	24	70.59
> =1000 ml	47	32.64	10	29.41
**Operation time**				
average (n/mean ± SD)	134	177.0 ± 54.3	43	166.2 ± 40.1
< 210 min (n/%)	101	75.37	35	81.40
> =210 min (n/%)	33	24.63	8	18.60
**Bone transplantation** (n/%)				
No	142	91.03	42	89.36
Yes	14	8.97	5	10.64
**Custom/ modular prosthesis** (n/%)				
Custom	91	58.33	31	65.96
Modular	65	41.67	16	34.04
**Prosthesis motion mode** (n/%)				
Rotational	137	87.82	40	85.11
Fixed	19	12.18	7	14.89
**Length of prosthetic stem**				
average (n/mean ± SD)	107	140.4 ± 23.8	43	136.3 ± 23.6
< 144 mm (n/%)	66	61.68	25	58.14
> =144 mm (n/%)	41	38.32	18	41.86
**Diameter of prosthetic stem**				
average (n/mean ± SD)	121	12.1 ± 1.7	43	11.9 ± 1.7
< 12 mm (n/%)	46	38.02	19	44.19
> =12 mm (n/%)	75	61.98	24	55.81
**Material of prosthesis** (n/%)				
Titanium	105	69.08	31	68.89
Vatallium	47	30.92	14	31.11
**Length of resection**				
average (n/mean ± SD)	150	12.8 ± 3.6	47	12.7 ± 3.7
< 10.4 cm (n/%)	40	26.67	14	29.79
> =10.4 cm (n/%)	110	73.33	33	70.21

Footnote: *Abbreviations: BMI* body mass index; *SD* standard deviation

Within 6 months post-operatively, a total of 26 (16.67%) and 7 (14.89%) patients were encountered with operation related complications, in the training and validation groups. The major complications leading to implant failure were aseptic loosening in 26 out of 20 patients (20/132, 15.1%), followed by superficial or periprosthetic infection (16), local recurrence or metastases or both (12), periprosthetic or prosthetic fracture (7), wound healing disorders (4), joint instability or flexion contracture in two (2), dislocation of the prosthesis in one patients (3). Four patients developed nonprosthesis-related complications include peroneal nerve palsy (2), others (3). Local relapse arose in 16 (10.26%) patients and 5 (10.64%) patients during the follow-up.

**Fig. 2:**
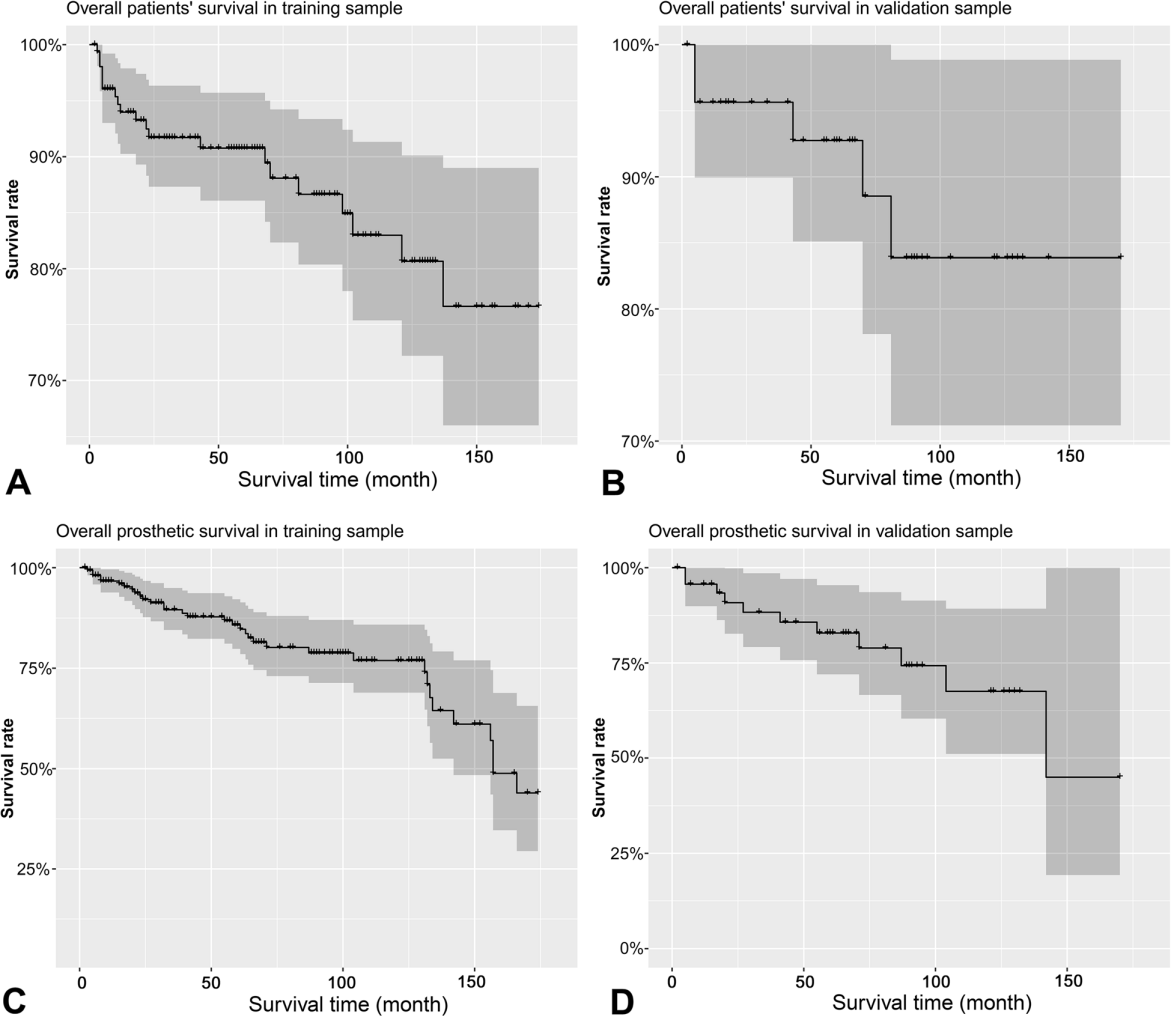
Kaplan-Meier survival curves for the patients’ overall survival and prosthetic survival in training and validation samples

The Kaplan-Meier survival curves for the patients’ overall survival and prosthesis survival are shown in Fig. 2. The overall patients’ survival at 12, 60, and 120 months were 94.0, 90.8 and 83.0%, and 95.7, 92.8 and 83.9% in training and validation groups, respectively. The prosthesis survival at 12, 60, 120, and 180 months were 96.7, 85.8, 76.9 and 44.0%, and 95.7, 82.9, 67.5 and 45.0% in training and validation groups, respectively.

### Results of univariate and multivariate COX analyses

**Fig. 3:**
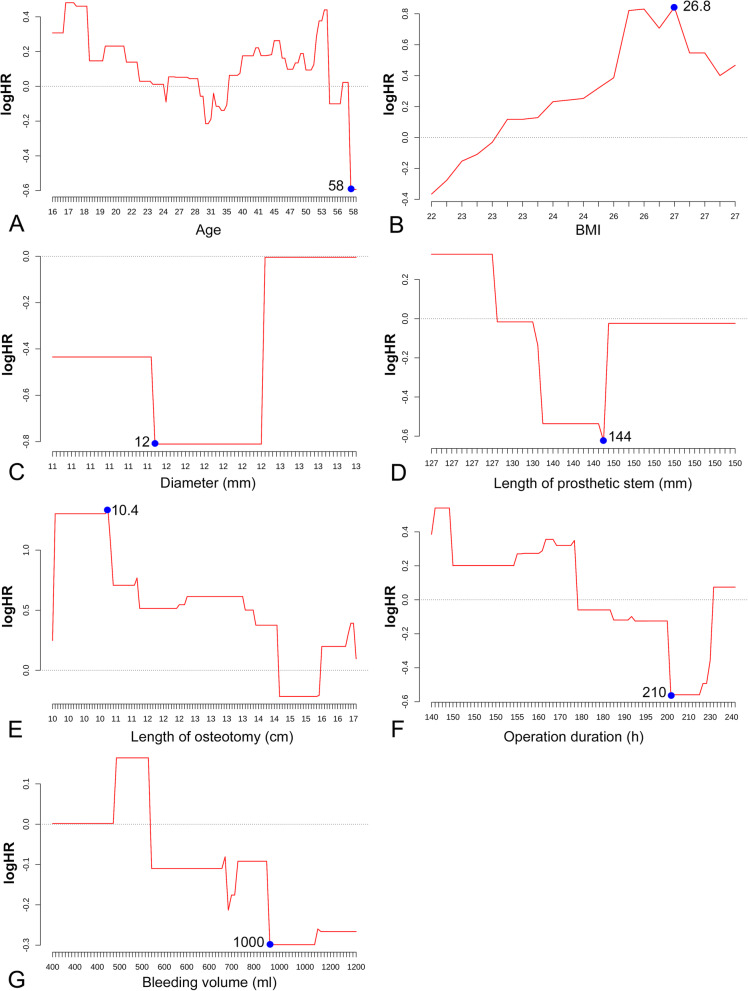
Results of running log-rank tests to detect the optimal cutoff points for continuous prognostic factors of implant survival, including age (**A**), BMI (**B**), stem diameter (**C**), stem length (**D**), length of osteotomy (**E**), operation duration (**F**) and bleeding volume (**G**)

**Fig. 4:**
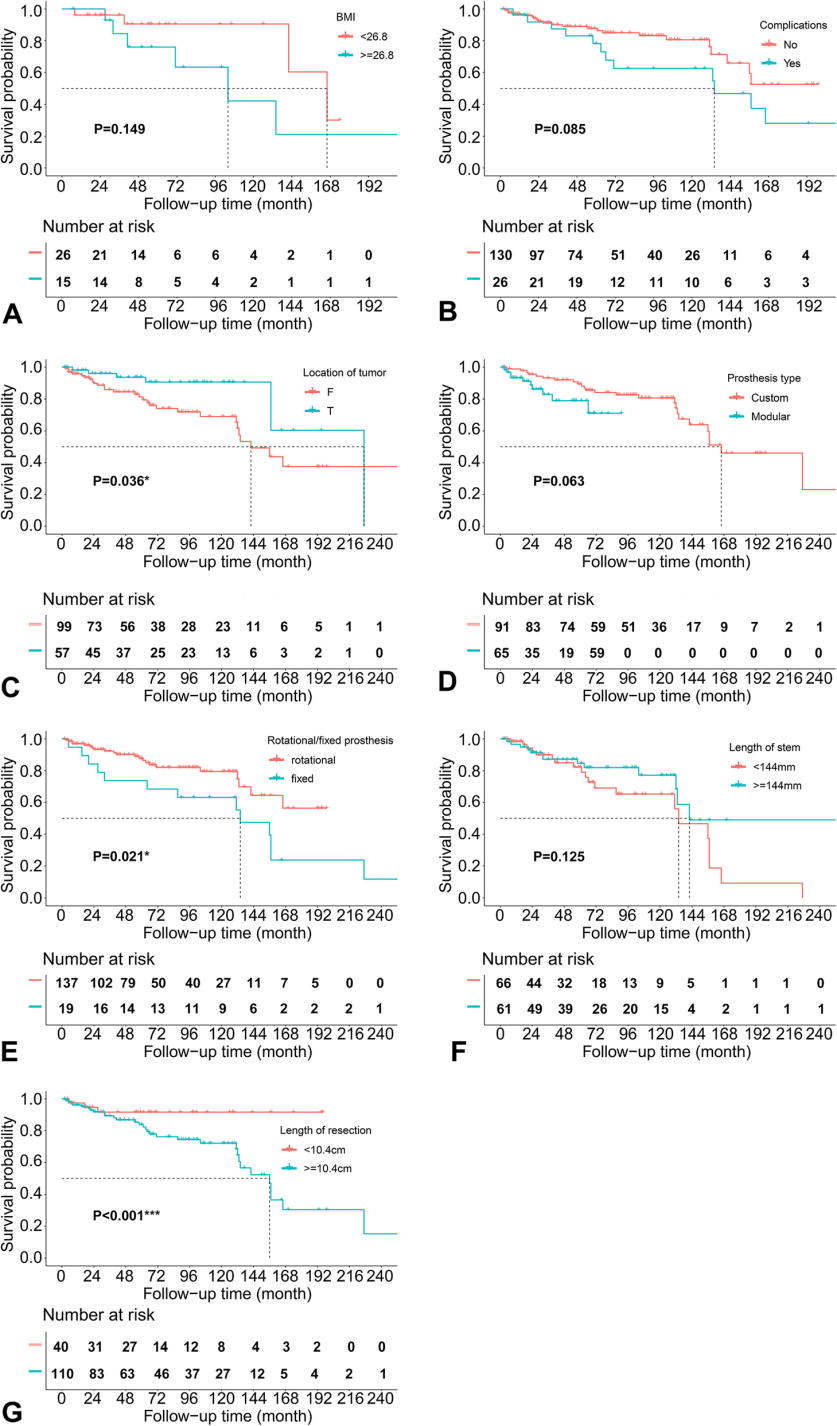
Kaplan-Meier survival curves for prognostic factors significantly associated with prosthetic survival, according to univariate COX analysis

Figure 3 shows the results of running log-rank test for the continuous predictors, presenting the optimal cut-off points to convert these factors into categorical variables. The results of univariate COX analysis are shown in Table 2, and the Kaplan-Meier survival curves for the significant and marginally significant (*p* < 0.15) prognostic factors are presented in Fig. 4. As a result, anatomical location at distal femur (HR = 0.412, 95%CI: 0.180 ~ 0.945, *P* = 0.036), fixed prosthetic motion mode (HR = 2.336, 95%CI: 1.134 ~ 4.812, *P* = 0.021), and length of resection (> = 10.4 cm; HR = 8.959, 95%CI: 3.767 ~ 21.310, *P* < 0.001) were significantly associated with increased implant failure, while BMI (HR = 2.555, 95%CI: 0.715 ~ 9.135, *P* = 0.149), operation related complications (HR = 1.877, 95%CI: 0.918 ~ 3.840, *P* = 0.085), custom/ modular prosthesis (HR = 2.244, 95%CI: 0.957 ~ 5.257, *P* = 0.063), and length of prosthetic stem (HR = 0.578, 95%CI: 0.287 ~ 1.165, *P* = 0.125) were demonstrated to be marginally associated with the survival of implant.

**Table 2:** Results of univariate COX analysis

Variables	Univariate COX analysis				
		HR	LCI	UCI	***P*** value
**Age**					
< 58 years	1				
≥ 58 years	1.021		0.358	2.913	0.969
**Sex**					
Male	1				
Female	0.937		0.463	1.898	0.857
**BMI**					
< 26.8	1				
≥ 26.8	2.555		0.715	9.135	**0.149**
**Tumor types**					
Borderline tumor	1				
Malignant primary tumor	0.718		0.358	1.438	0.350
Malignant metastatic tumor	0.000		0.000	Inf	0.997
**Operation related complications**					
No	1				
Yes	1.877		0.918	3.840	**0.085**
**Adjuvant chemotherapy/ radiotherapy**					
No	1				
Yes	1.058		0.545	2.057	0.867
**Pathological fracture**					
No	1				
Yes	1.479		0.731	2.993	0.277
**Primary/ revision reconstruction**					
Primary reconstruction	1				
Revision reconstruction	1.038		0.514	2.096	0.918
**Anatomical location**					
Distal femur	1				
Proximal tibia	0.412		0.180	0.945	0.036*
**Site of knee**					
Left	1				
Right	0.878		0.445	1.730	0.706
**Bleeding volume**					
< 1000 ml	1				
> =1000 ml	0.906		0.423	1.941	0.800
**Operation time**					
< 210 min	1				
> =210 min	0.881		0.368	2.105	0.775
**Bone transplantation**					
No	1				
Yes	1.506		0.622	3.645	0.364
**Custom/ modular prosthesis**					
Custom	1				
Modular	2.244		0.957	5.257	**0.063**
**Prosthesis motion mode**					
Rotational	1				
Fixed	2.336		1.134	4.812	0.021*
**Length of prosthetic stem**					
< 144 mm	1				
> =144 mm	0.578		0.287	1.165	**0.125**
**Diameter of prosthetic stem**					
< 12 mm	1				
> =12 mm	0.618		0.289	1.323	0.215
**Material of prosthesis**					
Titanium	1				
Vatallium	1.128		0.370	3.411	0.832
**Length of resection**					
< 10.4 cm	1				
> =10.4 cm	8.959		3.767	21.310	< 0.001**

*p < 0.05, **p < 0.001. *Abbreviations: HR* hazard ratio; *LCI* lower confidence interval; *UCI* upper confidence interval; *BMI* body mass index

**Fig. 5:**
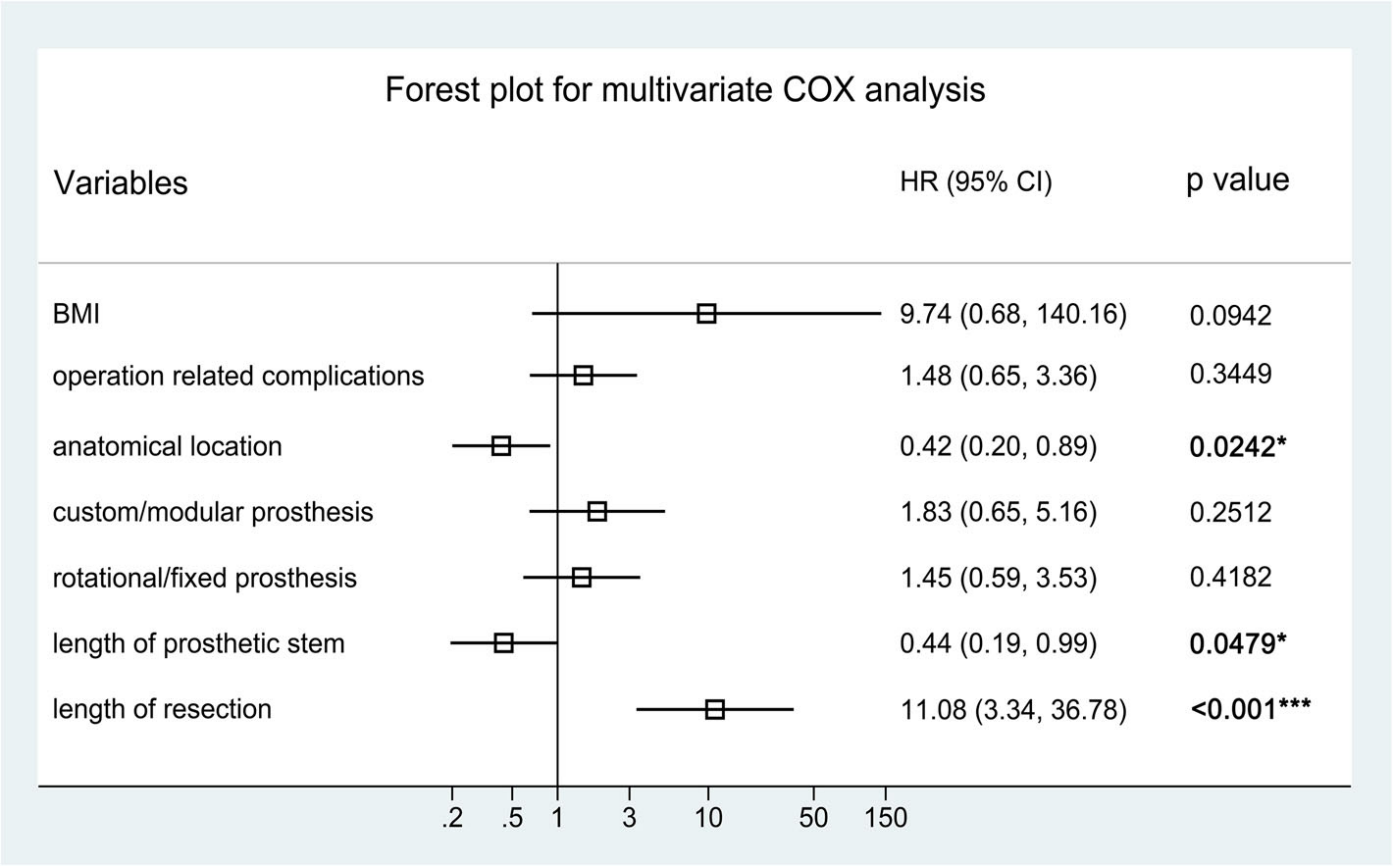
Forest plot displaying the effect sizes of prognostic factors involved in multivariate COX analysis

The forest plot in Fig. 5 shows the effect sizes of multivariate analysis, presenting that anatomical location (HR = 0.42, 95%CI: 0.20 ~ 0.89, *P* = 0.024), length of prosthetic stem (HR = 0.44, 95%CI: 0.19 ~ 0.99, *P* = 0.048) and length of bone resection (HR = 11.08, 95%CI: 3.34 ~ 36.78, *P* < 0.001) remained to be statistically significant prognostic factors for prosthetic survival.

### Establishing and validation of nomogram model

**Fig. 6:**
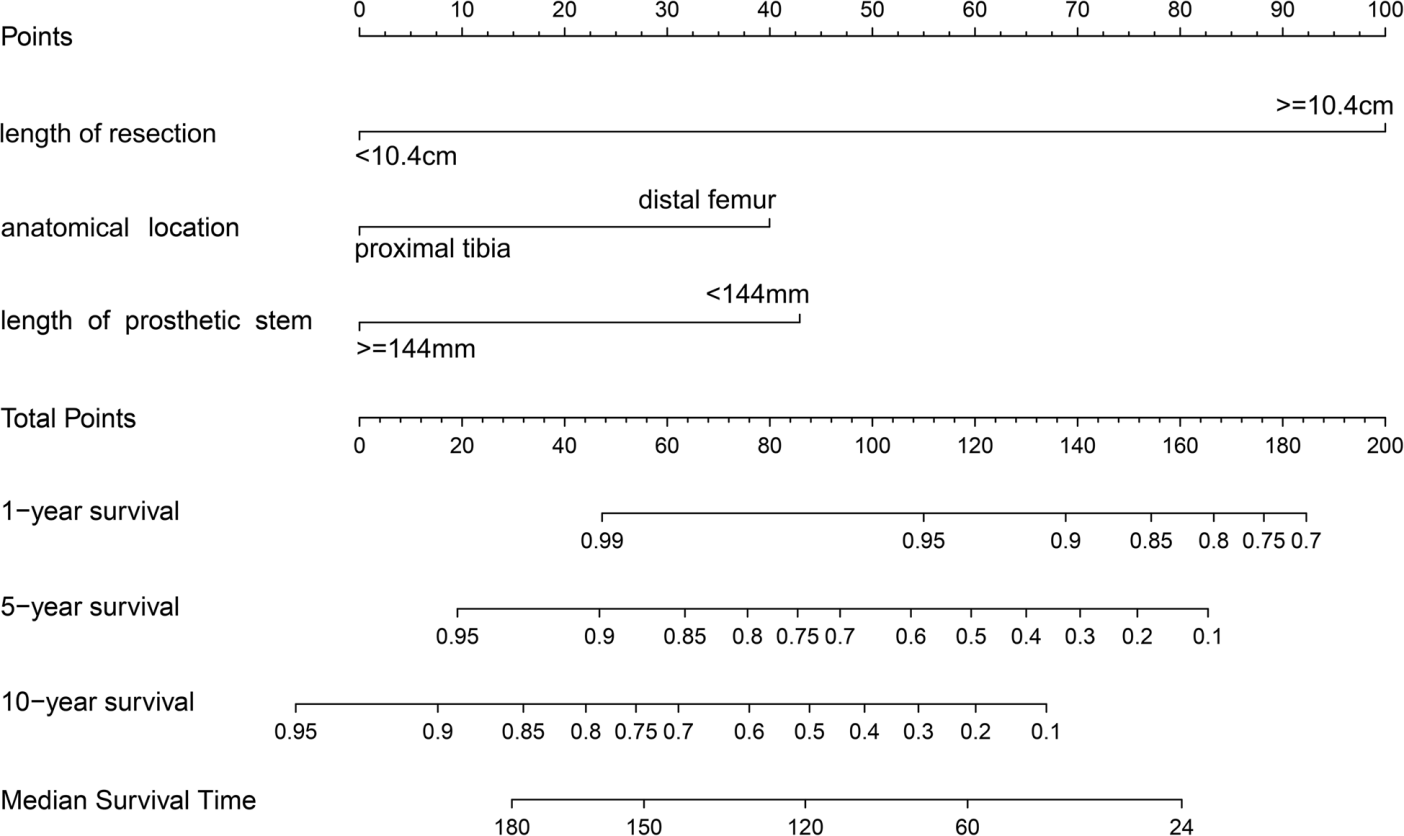
Graphical nomogram established for predicting the prosthetic survival at 1, 5 and 10 years, and the median prosthetic survival

Basing on the significant prognostic factors screened by multivariate COX model, a graphical predicting nomogram model was developed, to calculate the predicted prosthetic survival probabilities at 1, 5, and 10 years post-operatively, as well as the median survival time (Fig. 6).

**Fig. 7:**
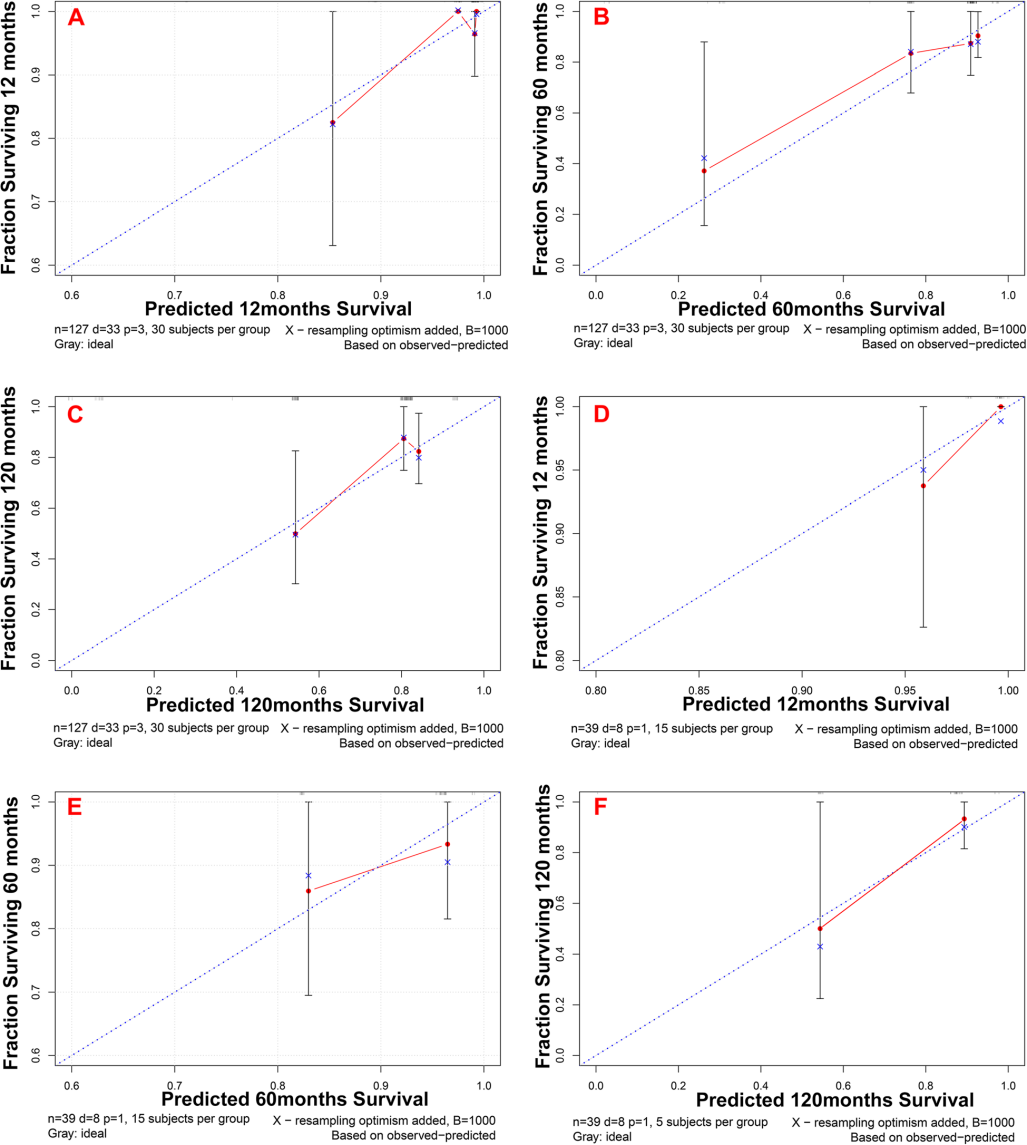
Calibration curves for evaluating the consistency between the predicted and actual survival probabilities, both in training and validation samples, at time points of 1, 5 and 10 years. Generally, satisfactory consistencies between predicted and actual survival rates were demonstrated

The C-indexes were 0.717 (95%CI: 0.497 ~ 0.937) and 0.726 (95%CI: 0.244 ~ 1.208) for the training and validation samples respectively, demonstrating that the newly established nomogram possesses favorable discrimination ability. The calibration curves at 12, 60 and 120 months for the two samples are displayed in Fig. 7. Generally, favorable consistencies between the predicted and actual survival were presented, indicating satisfactory accuracy of the novel predicting model.

To facilitate the usage of our novel predicting model, a web link (https://hellonihao.shinyapps.io/PangCG_DynNomapp/) was generated to automatically calculate the prosthetic survival online for each individual patient. The predicted survival curve, forest plot presenting survival probability, and numeric summary table would be output, after selecting of the patients’ characteristics and clicking on the button “Predict”. The survival probability at any time point could be obtained by dragging the sliderbar “time”.

## Case presentation

**Fig. 8:**
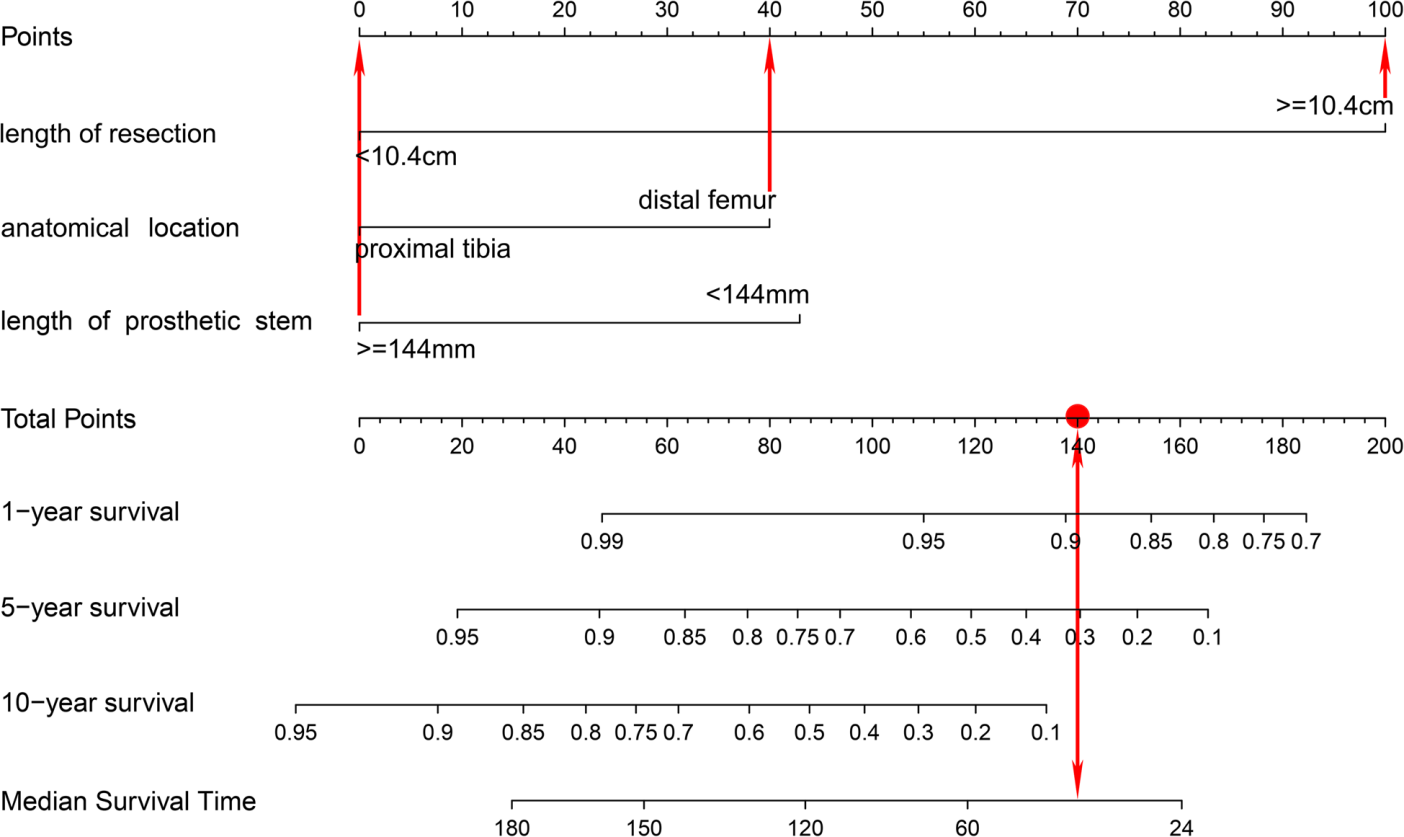
A case of patient who was operated for bone tumor locates at distal femur and reconstructed with a prosthesis that was more than 14.4 cm on length of stem, and more than 10.4 cm of bone was resected during tumorectomy. Using the graphical nomogram, survival rates could be read on the plot by drawing a vertical line through the total point

**Fig. 9:**
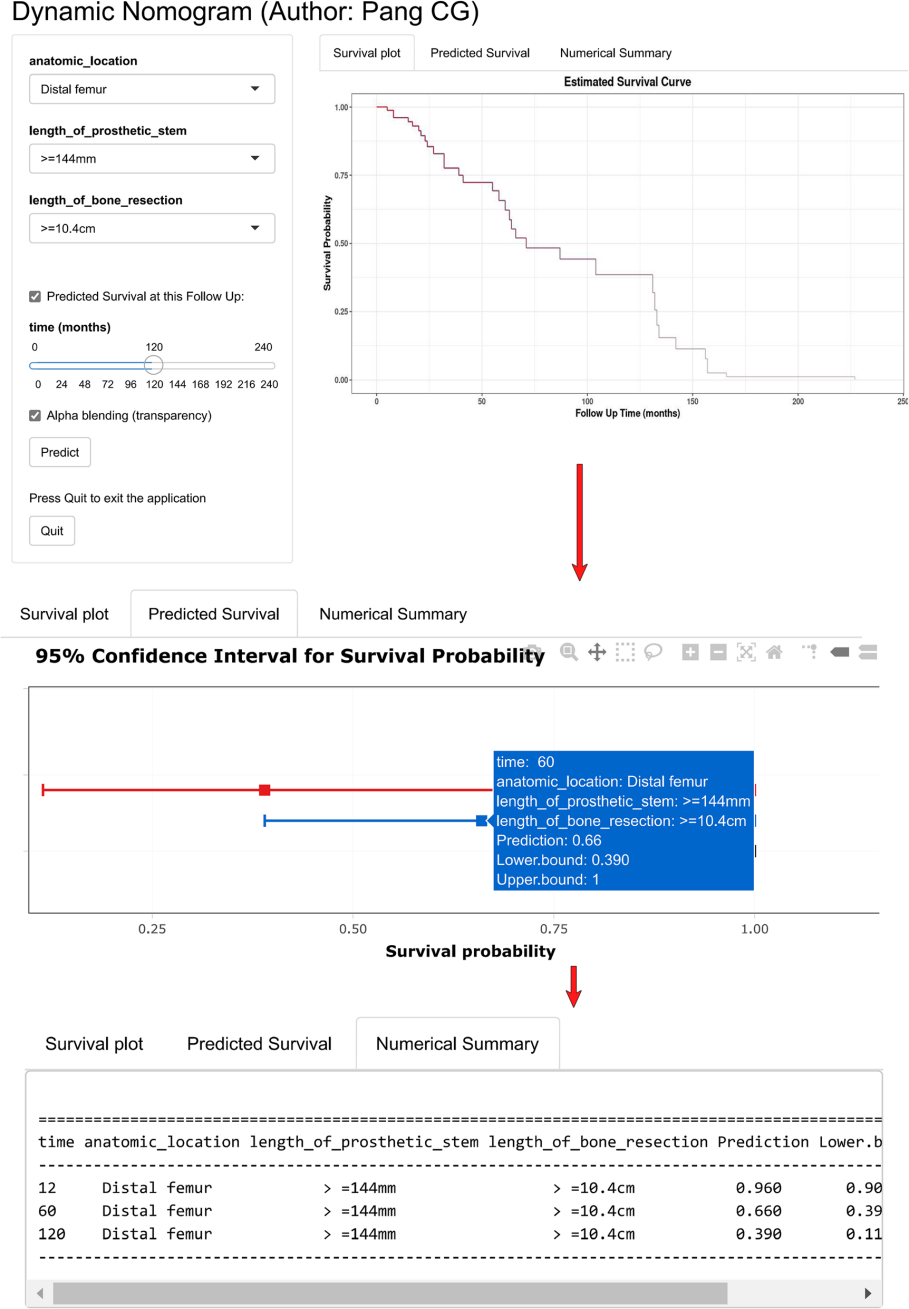
A case of patient who was operated for bone tumor locates at distal femur and reconstructed with a prosthesis that was more than 14.4 cm on length of stem, and more than 10.4 cm of bone was resected during tumorectomy. Through the webpage-based dynamic nomogram, the detailed prosthetic survival probabilities at 12, 60 and 120 months could be automatically calculated, which is shown to be 0.890, 0.305 and 0.068, respectively

Figure 8 and Fig. 9 show a case of patient who was operated for bone tumor locates at distal femur and reconstructed with a prosthesis that was more than 14.4 cm on length of stem, and more than 10.4 cm of bone was resected during tumorectomy. Using the graphical nomogram, only approximate survival rates could be read on the plot by drawing a vertical line through the total point. While, through the webpage-based dynamic nomogram, the detailed prosthetic survival probabilities at 12, 60 and 120 months could be automatically calculated, which is shown to be 0.890, 0.305 and 0.068, respectively.

## Discussion

Multimodality limb salvage therapy has replaced amputation as the preferred form of treatment for primary musculoskeletal tumors primarily owing to improvements in adjuvant and neo-adjuvant therapy, as well as advances in imaging and diagnostic modalities [2–4]. After the resection of bone and soft tissue tumors, reconstruction of the resulting defects was challenging because of the unique anatomic features of the knee. Limb-salvage surgery for malignant bone tumors must be successful in three different respects, that is, the cure of oncology, the durability of prostheses and the satisfactory of the function in the limbs. Following reconstruction of the operated knee, arising of complications in implants was not uncommon, and the prosthetic failure has always been regarded as a major event causing serious adverse effect on the patients’ overall survival, lower limb function and the quality of life, for these tumor patients. Thus, it is necessary to figure out the significant prognostic factors for prosthetic survival in patients operated for tumor around the knee. In the current study, we identified that the anatomical location of tumor, length of prosthetic stem, and length of bone resection during operation were significantly associated with the prosthetic survival. Basing on these factors, we also generated a graphic nomogram and a web predicting tool, which could easily applied for predicting survival probabilities of the implant at several time points.

Until now there are only a few predicting models that have been developed to help estimate the prosthetic survival probability, using independent prognostic factors. In the studies of Zhang et al. [19], they identified the significant factors associated with the incidence of aseptic loosening after tumor prosthetic replacement around the knee, demonstrating that tumor anatomical location (proximal tibia vs. distal femur), length of prosthetic stem (< 14 cm vs. > = 14 cm), and prosthetic motion mode (fixed hinge vs. rotation hinge) were independent predictors. After that, they established a graphical nomogram to help predict the risk of aseptic loosening at the time points of 5 and 10 years. This model, however, could only provide approximate risks of aseptic loosening at two points, and the applying of the model is not convenient as one need to calculate the sub-scores of the prognostic factors and the total score, and to gain the predicted survival by drawing vertical line. In the current study, we also developed a web-page based dynamic nomogram, to output a continuous survival curve, a forest plot of predicted survival and a numeric table online, being easier-to-use for clinicians and patients.

Regarding to the prognostic factors involved in our novel model, they have been widely analyzed in the previous researches [5, 20–23]. The anatomical location of the tumor at proximal tibia has been demonstrated to be associated with increased risk of implant failure compared to the tumor at distal femur in many former studies [5, 21–23]. In the study of Pala et al. [20], they reported that the failure rates of distal femoral and proximal tibia replacements were 26.7 and 36.7%, but the difference was non-significant between two groups. Hu et al. [5] also reported that the anatomical site of tumor was associated with the implant survival of tumor prosthesis around knee. Zhang et al. [21] investigated the prognosis value of site of the implant, and they reported a slight difference on the 5-year prosthetic survival (86.1% at distal femur and 66.9% at proximal tibia, *p* = 0.09). Guo et al. [22] identified the independent risk factors for implant failure in patients operated for tumor around the knee, and the result of multivariate COX analysis presented that the prosthesis implanted at the proximal tibia was associated with increased failure risk, when compared to that of the distal femur. Mazaleyrat et al. [23] reported that the 5-year and 10-year survival rates of implant were 84 and 70% for tumor located at distal femur, while 74 and 43% for tumor located at proximal tibia, being significantly different between the two groups by log-rank test (*p* = 0.02). Our result about the prognostic effect of anatomical location of tumor was in accordance with these previous researches. It could be speculated that the poor soft tissue coverage, difficulties with anchoring the patellar tendon and possible injuries to the neurovascular system are the most likely causes for this difference.

Length of prosthetic stem was another important predictor for the implant failure, in several published articles [19, 24, 25]. Bergin et al. [24] evaluated the influences of the diameter of prosthetic stem and length ratio of bone:stem on the incidence of aseptic loosening in distal femoral endoprostheses, and they found that patients with stable implants had significantly larger stem sizes and lower bone:stem ratios than those with loose implants. Zhang et al. [19] established a predicting nomogram for aseptic loosening after tumor prosthetic replacement around the knee, and they identified that the length of the prosthetic stem was independent prognostic factor, being included in their novel prediction model. This suggests that longer stem of the endoprostheses may have a mechanical advantage by obtain more osteointergration between the bone and the endoprosthesis, to prevent the early failures.

Resection length during tumorectomy was also recognized as an independent prognostic factor for prosthetic survival in former studies [5, 22, 24, 26, 27]. Guo et al. [22] found that a resection length of 14 cm or more was significant negative prognostic factor for endopristhetic replacement for primary tumors around the knee. Hu et al. [5] reviewed the published articles about application of artificial prosthesis reconstruction techniques in malignant tumors around the knee joint, showing that the longer the neoplastic bone resected, the worse the prosthetic survival rate is. Wu et al. [26] identified the prognostic effect of several factors, including the patient age, surgical stage, design of implant (custom vs. modular), stem diameter, and resection length, through multivariate COX analysis, demonstrating that design of prosthesis and length of resection were significantly associated with prosthetic survival. Kawai et al. [27] reported 82 cases of artificial prosthesis reconstruction after resection of distal femur malignant bone tumors. The prosthetic survival rate of the group in whom < 40% of the distal femur length was resected was considerably higher than that of the group with resection lengthmore than 40%, according to multivariate analysis. In our study, we divided the patients into two group by the cutoff point detected with running log-rank test, and found that patients with more than 10.4 cm of resection length was associated with increased implant failure. The influence of the extent of bone resections on the development of implant failure may be contributed by the increased torque production out of the line of prosthesis and/or impairment of quadriceps contraction. Changes in biomechanical stresses after extensive resections of bone and adjacent muscles probably are the reasons for the increased failure incidence of these implants.

This study, nevertheless, is of several limitations. Firstly, this study was designed as a retrospective cohort study, causing inevitable risk of bias on collection of the data. Thus, some more prospective and long-term cohort studies or randomized controlled trials should be conducted to clarified the prognostic effect of the predicting factors identified in this study. Secondly, as only a few patients were provided with uncemented prostheses in our institutions, it was impossible to take full assessment on the prognostic effect of the fixation method for the endoprostheses. Thus, we excluded patients reconstructed by implants without cement fixation to minimize the risk of bias caused by fixation methods. Thirdly, the patients used for external validation was sampled from the same cohort as the training sample used for model developing. Hence, further external validation using sample from different clinical centers or even different countries is necessary. Finally, it is difficult to collect large sample size in this topic due to the low frequency of tumor resection and endoprosthetic reconstruction for tumor around knee. Thus, to ensure the statistical power in the regression analysis and model developing, most participants were put in the training sample, causing the relative small size of validation sample.

## Conclusions

The anatomical location of the endoprostheses, length of the prosthetic stem and the length of the bone resection were demonstrated to be significantly associated with the risk of implant failure in patients reconstructed for tumors around the knee. A graphical nomogram and a user-interactive online dynamic nomogram were developed to help clinicians and patients easily predict the survival probabilities of implant at any time point post-operatively.

## Supplementary Information

**Additional file 1.**

## Data Availability

The datasets used and/or analyzed during the current study are available from the corresponding author on reasonable request.

## Abbreviations

CPM: continuous passive motion
BMI: body mass index
CI: confidence interval
HR: hazard ratio
MRI: magnetic resonance imaging
MSCT: Multi Slice Computerized Tomography
MSTS: Musculoskeletal Tumor Society
TRIPOD: Transparent Reporting of a Multivariable Prediction Model for Individual Prognosis or Diagnosis
